# Determination of Urinary Neutrophil Gelatinase-Associated Lipocalin (uNGAL) Reference Intervals in Healthy Adult and Pediatric Individuals Using a Particle-Enhanced Turbidimetric Immunoassay

**DOI:** 10.3390/diagnostics15010095

**Published:** 2025-01-03

**Authors:** Tabari M. Baker, Christopher A. Bird, Dennis L. Broyles, Ursula Klause

**Affiliations:** 1 BioPorto A/S, 2900 Hellerup, Denmark; tmb@bioporto.com (T.M.B.); christopherabird@gmail.com (C.A.B.); 2 L3 Healthcare, San Diego, CA 92101, USA; dennis.broyles@covarsadx.com

**Keywords:** biomarker, acute kidney injury, reference range interval, neutrophil gelatinase-associated lipocalin

## Abstract

**Background**: The current gold standards for diagnosing acute kidney injury (AKI) are an increase in serum creatinine and a decrease in urine output, which are inadequate for rapid diagnosis. Neutrophil gelatinase-associated lipocalin (NGAL) is a 25-kDa protein produced and secreted by injured kidney tubule epithelial cells, and can serve as an early urinary biomarker for AKI. ProNephro AKI (NGAL) is an immunoassay for the quantitative determination of NGAL in urine (uNGAL) that recently received FDA clearance. A multisite, cross-sectional study was conducted to establish reference intervals for uNGAL in apparently healthy individuals. **Methods**: Urine samples were collected from apparently healthy individuals aged ≥3 months who met all inclusion criteria and no exclusion criteria. Specimens were temporarily stored at room temperature or 2–8 °C, then transferred into urinalysis tubes before being frozen and shipped for testing. uNGAL testing was performed using the ProNephro AKI (NGAL) immunoassay on a Roche cobas c501 analyzer. **Results**: Of the 688 individuals screened, 677 were eligible, and 629 (91.4%) of those were deemed evaluable. The 95th and 97.5th percentile uNGAL values for all pediatric participants were below the clinical cutoff of 125 ng/mL. uNGAL values were statistically significantly higher for female vs. male participants in both adult (*p* = 0.003) and pediatric groups (*p* < 0.001), while differences were not statistically significant for age, site location, race, or ethnicity. **Conclusions**: This study provides normal reference intervals for uNGAL with the ProNephro AKI (NGAL) clinical chemistry immunoassay that may be useful for interpreting patient results.

## 1. Introduction

Acute kidney injury (AKI) is a challenging condition characterized by a sudden onset of kidney dysfunction [1,2]. It is not technically a single disease, but a heterogeneous syndrome with various clinical presentations and potential etiologies, which include kidney hypoperfusion, cardiac dysfunction, and intra-abdominal hypertension [3]. AKI occurs most commonly after surgery or critical illness [2] and affects approximately 20% of hospitalized adults [4], 20–30% of children and adolescents admitted to the pediatric intensive care unit (PICU) [5], and up to 50% of pediatric patients who undergo cardiac surgery [1].

AKI is associated with serious sequelae, including higher rates of renal-replacement therapy and mechanical ventilation, prolonged hospital and ICU stays, and increased long-term mortality [5,6,7]. One study of pediatric patients admitted to a PICU found that AKI was independently associated with more than tripling patients’ mortality risk within 5–7 years of discharge [8]. Furthermore, research suggests that children who have severe AKI or multiple AKI episodes are more likely to experience long-term complications like hypertension, reduced kidney function, and progressive chronic kidney disease or end-stage renal disease requiring dialysis or transplantation [9,10].

About one-third of all AKI cases are present at hospital admission or develop within 24 h [11], and the likelihood of poor outcomes increases as severity progresses [12]. Therefore, early detection of AKI is essential, as it can help clinicians take early action that may help reverse subclinical kidney cell injury and avoid permanent renal damage [3,13]. But, a recognized, universal tool for early detection is not yet available [2], and current diagnostic methods preclude immediate intervention [3,9].

The gold standard methods for AKI diagnosis at this time are an increase in serum creatinine and a decrease in urine output [14], but these functional biomarkers are limited by delayed results and their low sensitivity and specificity, making them insufficient for quickly and accurately diagnosing AKI [3,6,15].

Creatinine is produced in the muscles at a constant rate and is typically cleared from the blood by the kidneys, with serum levels rising when clearance decreases [16]. This makes serum creatinine an indirect measure of kidney function that fluctuates based on muscle mass and other factors unrelated to renal disease (e.g., age, race, gender, diet, and hydration status) [16,17]. Serum creatinine also does not rise until 48–72 h after injury and some research suggests that about 50% of renal function must be lost before levels increase [6,16]. Another shortcoming is the inability to detect structural tubular injury that occurs without a rise in serum creatinine levels (i.e., subclinical AKI) [6], with one study finding that 21% of patients with acute tubular injuries would have been missed if serum creatinine alone was used for the diagnosis [18].

These limitations suggest that serum creatinine is not adequate for the timely diagnosis and subsequent treatment response for AKI, as therapy is only effective if administered soon after injury and before serum creatinine levels begin to rise [17]. In 2020, the Acute Disease Quality Initiative (ADQI) workgroup recommended that “a combination of damage and functional biomarkers, along with clinical information, be used to improve the diagnostic accuracy of AKI, to recognize the different pathophysiological processes, to discriminate AKI etiology, and to assess AKI severity [15]”.

Several novel biomarkers have emerged that can detect AKI earlier and with better sensitivity than serum creatinine [15]. Among these is neutrophil gelatinase-associated lipocalin (NGAL), a 25-kDa protein found in many human tissues, including kidney tubular epithelial cells [3,19]. NGAL was first identified as a novel protein isolated from secondary granules of human neutrophils [20]. Subsequent research with genomic and proteomic technology found elevated NGAL levels in mouse models of ischemia-reperfusion injury, showing that NGAL was a molecular indicator of epithelial injury [21]. Later studies revealed that NGAL levels rose significantly in children with AKI after cardiopulmonary bypass [22,23], and these findings were then confirmed in adults, in which NGAL increased substantially hours after cardiac surgery [24].

NGAL is typically expressed in low concentrations, but levels rise rapidly after kidney injury [3,19]. Studies have shown that changes in NGAL levels—which can be measured in urine or plasma—occur 2 h after kidney injury and 2–3 days before serum creatinine rises [6,25]. This provides the advantage of identifying patients with subclinical AKI who may be overlooked based on serum creatinine levels, such as 43% of patients in one study diagnosed with AKI using NGAL who would not have been diagnosed using serum creatinine alone [13]. Both biomarkers can also be used together, such as when a negative urinary NGAL (uNGAL) with a positive serum creatinine suggests a patient who has reversible AKI with no structural tubular damage [14].

The NGAL biomarker has been investigated extensively in numerous settings and populations, with more than 2500 animal and human studies published [26]. Several meta-analyses have shown that NGAL, either alone or with creatinine, is a useful biomarker that identifies AKI accurately and quickly in pediatric patients [2,27,28]. One meta-analysis found that the uNGAL-to-creatinine ratio has the best odds for diagnosing AKI, with a sensitivity of 91.3% and specificity of 89.7% [27]. NGAL was also found to have good diagnostic ability to predict both AKI risk and mortality in children after cardiac surgery and was ranked as the best biomarker for recognizing AKI early (within 2–6 h of kidney injury) [28].

ProNephro AKI (NGAL) (BioPorto Diagnostics A/S.; Hellerup, Denmark) is a particle-enhanced turbidimetric assay for the quantitative determination of uNGAL on automated clinical chemistry analyzers. The assay was FDA 510(k) cleared on 7 December 2023, and is the first AKI biomarker test in the US available for use in pediatric patients aged 3 months through 21 years [29]. ProNephro AKI (NGAL) is intended to help clinicians diagnose moderate-to-severe AKI and identify at-risk patients in the ICU within 48–72 h, thereby enabling prompt intervention [29].

To adhere to FDA submission requirements and improve the clinical utility of ProNephro AKI (NGAL), normative uNGAL values in healthy individuals need to be established [17]. Therefore, a reference interval study was conducted to report uNGAL levels in apparently healthy populations comprised of various ages, sexes, and racial and ethnic groups from different geographic locations in the US.

## 2. Materials and Methods

### 2.1. Study Design

This was a multisite, cross-sectional reference interval study conducted to collect and analyze urine samples from apparently healthy pediatric and adult individuals with the ProNephro AKI (NGAL). The primary objective was to establish uNGAL reference range intervals.

The protocol received unconditional approval from the Western-Copernicus Group Institutional Review Board, and the study was conducted in accordance with the ethical principles originating in the Declaration of Helsinki. All participants provided written informed consent or assent, if necessary.

### 2.2. Investigational Device Description

ProNephro AKI (NGAL) is a particle-enhanced turbidimetric immunoassay for the quantitative determination of uNGAL on automated clinical chemistry analyzers. It requires a prescription and is intended to be used in conjunction with clinical evaluation in pediatric patients aged ≥3 months to <22 years without underlying kidney disease admitted to the ICU for cardiovascular or respiratory compromise or who have had a solid organ or bone marrow transplant. ProNephro AKI (NGAL) is intended to be used on a mid-volume analyzer (cobas c 501 module; Roche, Indianapolis, IN, USA) by laboratory professionals in a Clinical Laboratory Improvement Amendments-certified laboratory in the first 24 h of ICU admission to aid risk assessment of moderate-to-severe AKI (Stage 2–3) 48–72 h after the assessment. A uNGAL value below the clinical cutoff of 125 ng/mL is considered a negative result and indicates a lower risk of developing or having moderate-to-severe AKI, while a value ≥ 125 ng/mL indicates a higher risk.

ProNephro AKI (NGAL) contains reaction buffer reagent (R1) and immunoparticle suspension reagent (R2). The R1 reagent is a ready-to-use tris-buffer solution containing murine protein and preservative. The R2 reagent is a ready-to-use suspension of polystyrene microparticles coated with mouse monoclonal antibodies to NGAL that also contains preservative. The ProNephro AKI (NGAL) Calibrator Kit consists of five individual ready-to-use calibrator solutions (1 mL each) comprised of different concentrations of recombinant human NGAL, ranging from 50–3000 ng/mL, in a HEPES (4-[2-hydroxyethyl]-1-piperazineethanesulfonic acid) buffer and a preservative. The ProNephro AKI (NGAL) Control Kit contains ready-to-use high (~500 ng/mL) and low controls (~200 ng/mL) comprised of recombinant human NGAL in HEPES buffer and a preservative. There are three bottles of each concentration level of the ready-to-use control solutions (1 mL each) included with each kit.

Once collected, each urine sample is processed and mixed with R1. After a short incubation, the reaction is started by adding R2. uNGAL in the sample causes the immunoparticles to agglutinate, and the degree of agglutination is quantified by light scattering, which is measured as absorption of light at a 570 nm wavelength. The absorbance change is read ~6 min after R2 is added, and the uNGAL concentration is determined by interpolating a calibration curve prepared from calibrators of known concentrations.

### 2.3. Participant Selection

Specimens were to be collected from individuals of geographically diverse locations to ensure a wide range of uNGAL values. The goal was to include a minimum of 600 healthy participants (300 males and 300 females), with ≥60 male and female participants in each age group: infants (≥3 months to <2 years), children (≥2 years to <12 years), adolescents (≥12 years to <22 years), adults (≥22 years to <65 years), and seniors (≥65 years).

Individuals aged ≥3 months who were apparently healthy and signed the written informed consent (or assent) form were eligible for inclusion. Individuals were excluded if they had a urinary tract infection (UTI), current or previous AKI, current or previous Stage 4 or 5 chronic kidney disease (CKD), known congenital anomalies of the kidney and urinary tract, or known urothelial, urological, or kidney malignancy. Additional exclusion criteria were uncorrected congenital heart disease (except those with isolated uncorrected ventricular septal defect, atrial septal defect, patent ductus arteriosus, or patent foramen ovale), current renal replacement therapy, history of nephrectomy or surgical correction of congenital heart disease within the previous 3 months, and history of a urologic procedure/surgery or solid organ, renal, or bone marrow transplantation. Individuals who were not able or willing to contribute the required urine specimen for testing were also excluded.

### 2.4. Specimen Collection and Testing

Individuals who met all inclusion criteria and no exclusion criteria were enrolled. Specimens were collected at 4 clinical enrollment sites—including mobile sites—located throughout the US (Escondido/Murietta, CA; Needham, MA; Indianapolis, IN; Bluffdale, UT). Enrolled participants provided clinical information, completed a questionnaire, and self-collected a 15 mL urine sample, with parents assisting their child(ren) as needed. Specimens were stored at room temperature for ≤4 h or at 2–8 °C for ≤48 h after collection, and were then transferred into urinalysis tubes prior to freezing. Samples were shipped to a central laboratory location and upon completion of the collection, shipped to the Bio-Porto Research and Development (R&D) laboratory (Hellerup, Denmark) for testing. Temperature logs were maintained and recorded.

uNGAL testing was performed at the BioPorto R&D laboratory with the ProNephro AKI (NGAL) assay and a mid-volume analyzer. Samples were tested randomized in single determinations.

UTI testing was performed on fresh samples at each collection site and also performed on thawed samples using reagent strips (Multistix 7; Siemens, Malvern, PA, USA). Samples were visually inspected following the product guide. Following published guidance [30], samples were excluded due to UTI if at least one of the following criteria was fulfilled: (1) moderate-to-high level of leukocyte esterase or (2) nitrile positive.

### 2.5. Statistical Analysis

All data was analyzed using the R statistical software, version 4.1 (R Core Team, 2021; R Foundation for Statistical Computing, Vienna, Austria) [31].

BioPorto maintained the testing line data. Testing data and results were checked and verified by BioPorto personnel to ensure alignment based on subject ID numbers, completeness and accuracy of data, and initial review of quantitative results. All source documentation was 100% source data verified, including all consent forms and case report forms.

Participants were classified by age, sex, and enrollment site. At least 120 participants per age group were targeted to calculate separate reference intervals. If partitioning (e.g., by sex) was not required, a single reference interval could be established. Reference interval limits were determined non-parametrically by estimating percentiles with the equation “*p* = (r − 0.5)/*N*”, with *p* as the percentile probability, r as rank, and *N* as sample size. This follows the recommendation for non-parametric percentile estimates for smaller samples [32] and allows the same non-parametric procedure to be used to estimate limits for both sex and age groups. It also follows the recommendation for non-parametric percentile estimates from the Clinical and Laboratory Standards Institute EP17-A2 [33].

Confidence intervals were calculated using non-parametric bootstrap resampling with 10,000 resamples used to generate 90% confidence intervals (CIs) around reference range limit estimates. Subgroup differences were tested with the Wilcoxon–Whitney rank sum test and the Kruskal–Wallis rank sum test. For variables of interest that were non-normally distributed (e.g., uNGAL), the non-parametric Wilcoxon–Whitney Rank Sum Test was used to statistically compare 2 independent groups (e.g., males vs. females). The non-parametric Kruskal–Wallis Rank Sum Test was used for ≥3 independent groups.

The assay measuring range (AMR) of the ProNephro AKI (NGAL) assay is 50 to 3000 ng/mL, as determined by the lowest and highest calibrator. The instrument reports all results of ≤50 ng/mL as “50” with a flag “<test”. Since a numerical value is required for statistical analysis, all such results were set to 50 ng/mL, although the true concentration of uNGAL in healthy individuals could be below this value.

## 3. Results

Enrollment took place from October 2020 through July 2021. There were 688 individuals screened for inclusion, 677 of whom were eligible and subsequently enrolled. Of these, 629 individuals (91.4%) were evaluable per the protocol, with the remaining 59 (8.6%) found to be ineligible after enrollment or not evaluable. Reasons for exclusion included meeting ≥1 exclusion criteria, an incorrect or incomplete informed consent form, or a positive UTI result. No modifications were made to the protocol, no protocol deviations were noted, and no adverse events occurred.

Baseline demographic information for the overall and pediatric populations is presented in Table 1. The pediatric population was comprised of participants aged ≥3 months to <22 years, and the youngest age group of ≥3 months to <2 years had the lowest number of participants (*n* = 73). There was a relatively even distribution of male and female participants in all age groups (Table 2).

The Wilcoxon–Whitney rank sum test with continuity correction identified a statistically significant difference in uNGAL values between male and female participants in both the adult population (*p* = 0.003) and pediatric population (*p* < 0.001), but this difference was not statistically significant for the overall population. No statistically significant differences were found for age, site location, race, or ethnicity with the Wilcoxon–Whitney rank sum test with continuity correction or Kruskal–Wallis rank sum test (all *p* > 0.05).

**Figure 1:**
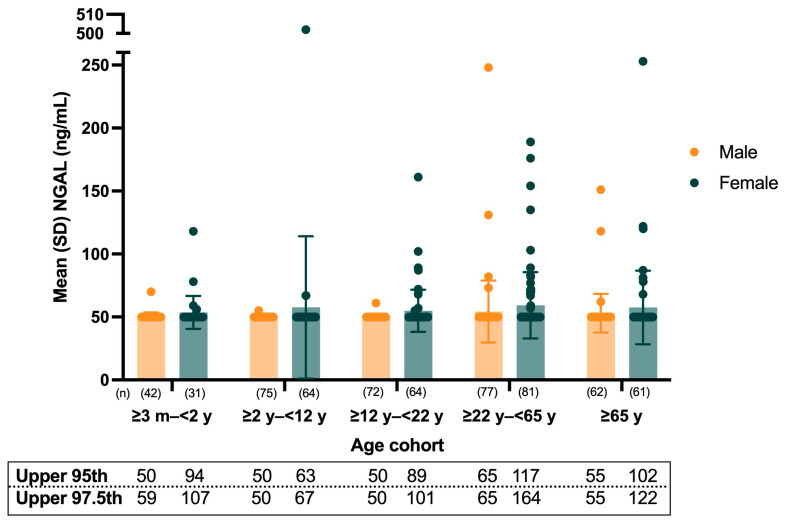
Summary statistics and individual uNGAL values for participants by age group. SD, standard deviation; uNGAL, urinary neutrophil gelatinase-associated lipocalin.

The upper 95th and 97.5th percentile uNGAL reference values for all participants are shown in Figure 1. For the entire pediatric population aged ≥3 months to <22 years, upper 95th percentile uNGAL values were 50 ng/mL (90% CI, 50–50 ng/mL) for male and 72 ng/mL (90% CI, 57–89 ng/mL) for female participants, while upper 97.5th percentile uNGAL values were 50 ng/mL (90% CI, 50–59 ng/mL) for male and 96 ng/mL (90% CI, 71–161 ng/mL) for female participants. Both the 95th and 97.5th percentile reference values for all pediatric age groups remained below the clinical cutoff of 125 ng/mL [34].

## 4. Discussion

This multisite, cross-sectional reference range study has established normal uNGAL values in apparently healthy children, adolescents, and adults obtained with the ProNephro AKI (NGAL) immunoassay. Specimens were collected from apparently healthy individuals of various ages, races, and ethnicities at 4 sites throughout the US with a relatively even distribution of males and females. The 95th and 97.5th percentile uNGAL values for all pediatric participants were below the clinical cutoff of 125 ng/mL, which indicates a negative result and would be expected in this healthy population.

The uNGAL value of 125 ng/mL was proposed as the optimal clinical cutoff for predicting Stage 2–3 AKI within 48–72 h of ICU admission in two coordinated studies of pediatric patients aged ≥90 days to <22 years [34]. This value was derived from uNGAL measurements of pediatric patients who were eventually diagnosed with AKI in the first study, and the cutoff of 125 ng/mL demonstrated excellent specificity and negative predictive value for Stage 2–3 AKI at 48–72 h in both studies.

Although differences in uNGAL values were identified for several groups, the only statistically significant difference was in sex, with female participants having higher uNGAL values than male participants in both pediatric and adult populations. This finding is consistent with reference intervals published elsewhere, with several studies reporting higher uNGAL values in female participants. One study of 174 healthy adults identified 95th percentile values of 129 ng/mL for females and 91 ng/mL for males (*p* = 0.007) [19], while another study of 368 healthy children found the 95th percentile cutoff values to be significantly higher for female (73.1 ng/mL) than male participants (28.3 ng/mL) [17]. Other studies have also demonstrated higher uNGAL values for females vs. males in several populations, including healthy newborns and children [35,36], healthy adults [37], and adult kidney transplant recipients [38].

The age-related differences in uNGAL values were not statistically significant or clinically relevant, but clinically relevant differences between age groups were not expected at the uNGAL concentrations detectable by the ProNephro AKI (NGAL) immunoassay. This is also comparable to uNGAL reference values found in related research, where values generally increase with age [17,19]. For example, one study found 95th percentile uNGAL cutoff values for healthy children of 39.1 ng/mL in ages 3 to <5 years, 58.5 ng/mL in ages 5 to <10 years, 66.8 ng/mL in ages 10 to <15 years, and 74.7 ng/mL in ages 15 to <18 years [17]. This is further supported by a meta-analysis of studies using NGAL to predict AKI, which established a cutoff of 170 ng/mL for adults vs. 100–135 ng/mL for children [39]. One exception may be neonates, who have significantly higher mean uNGAL values (44.2 ng/mL) than children (10.2 ng/mL) (*p* < 0.0001) [35], which may be due to the immature development of the kidney at this age [17].

Other variables that could potentially influence uNGAL levels include height and weight, which are related to renal length and mass [17], but our study was not designed to analyze these parameters.

This study does have some limitations. As previously stated, uNGAL values < 51 ng/mL were below the lower limit of the AMR and were entered into the analyses as 50 ng/mL. Many of the true uNGAL concentrations for participants were below this level, with some likely having values between 10–30 ng/mL. This may have affected the veracity of these uNGAL reference ranges, but obtaining results under the lower limit of the AMR was expected since the population studied was apparently healthy. We must also note that the number of participants in the youngest age group (*n* = 73) was lower than other groups and did not meet our target goal of 120 participants per age group. This was due to recruitment issues and some difficulty using urine collection bags with very young children. We recognize this limitation. However, this would be a real-world limitation in clinical practice, and this patient population would demonstrate increased susceptibility to AKI. Our data suggests that the reference interval for uNGAL in the youngest population is well-below a clinical cutoff, and though enrollment did not meet our target goal in this age group, can inform clinicians of an appropriate range for uNGAL in this vulnerable, understudied population. Lastly, since participants self-reported their condition as “healthy”, it is possible that some had an asymptomatic kidney disease unbeknownst to them, which may have produced slightly elevated uNGAL values.

Future studies should investigate clinical cutoffs for uNGAL values in healthy adults aged ≥22 years and specific patient populations, such as patients admitted to a hospital through the emergency department, adults who have undergone a cardiac procedure or solid organ transplant, and adults with a systemic bacterial infection or sepsis. Studies to establish NGAL reference ranges in plasma and serum are also needed.

## 5. Conclusions

In conclusion, this is the largest comprehensive study to date to establish uNGAL reference range values with the ProNephro AKI (NGAL) immunoassay in apparently healthy children, adolescents, and adults. We demonstrate that the reference values for all patient populations fell below the clinical cutoff. Across the study population, uNGAL values for female participants were found to be significantly higher than for male participants, similar to previous reports. These data serve as a basis for establishing the clinical utility of ProNephro AKI (NGAL) in the assessment of AKI risk in various patient populations.

## Figures and Tables

**Table 1:** Baseline demographic information.

Parameter	Overall Population (*N* = 629)	Pediatric Population (*N* = 348)
Age, mean ± SD, y	29.7 ± 26.2	9.3 ± 6.0
Median	17.3	9.2
Range	0.26–89.5	0.26–21.7
Male sex, *n* (%)	328 (52.1)	189 (54.3)
Race, *n* (%)		
American Indian/Alaskan Native	4 (0.6)	0 (0)
Asian	29 (4.6)	16 (4.6)
Black/African American	18 (2.9)	12 (3.4)
Native Hawaiian or Pacific Islander	2 (0.3)	0 (0)
White	541 (86.0)	303 (87.1)
Unknown/prefer not to answer	8 (1.3)	6 (1.7)
Other	27 (4.3)	11 (3.2)
Ethnicity, *n* (%)		
Hispanic/Latino	67 (10.7)	36 (10.3)
Not Hispanic/Latino	554 (88.1)	306 (87.9)
Not reported	8 (1.3)	6 (1.7)

SD, standard deviation.

**Table 2:** Number of participants in each age group (overall population; *N* = 629).

	Female *n* (%)	Male *n* (%)	Total
Overall population	301 (47.9)	328 (52.1)	629
≥3 months to <2 years	31 (42.5)	42 (57.5)	73
≥2 years to <12 years	64 (46.0)	75 (54.0)	139
≥12 years to <22 years	64 (47.1)	72 (52.9)	136
≥22 years to <65 years	81 (51.3)	77 (48.7)	158
≥65 years	61 (49.6)	62 (50.4)	123

## Data Availability

Data will be made available on request.
